# Axonal polyneuropathy and ataxia in children: consider Perrault Syndrome, a case report

**DOI:** 10.1186/s12920-023-01599-4

**Published:** 2023-11-06

**Authors:** Hannah E. Munson, Lenika De Simone, Abigail Schwaede, Avanti Bhatia, Divakar S. Mithal, Nancy Young, Nancy Kuntz, Vamshi K. Rao

**Affiliations:** 1 Chicago College of Osteopathic Medicine, Midwestern University, Downers Grove, IL USA; 2 Division of Genetics, Birth Defects and Metabolism, Ann and Robert H. Lurie Children’s Hospital of Chicago, Chicago, IL USA; 3 Division of Neurology, Department of Pediatrics, Northwestern University Feinberg School of Medicine and the Ann and Robert H. Lurie Children’s Hospital of Chicago, Chicago, IL USA; 4 Department of Speech-Language Pathology, Ann and Robert H. Lurie Children’s Hospital of Chicago, Chicago, IL USA; 5 Stanley Manne Children’s Research Institute, Chicago, IL USA; 6 Division of Otolaryngology, Department of Otolaryngology, Ann and Robert H. Lurie Children’s Hospital of Chicago, Northwestern University Feinberg School of Medicine, Chicago, IL USA

**Keywords:** Perrault Syndrome, PRLTS, TWNK, Ataxia, Axonal polyneuropathy, Auditory neuropathy/auditory synaptopathy

## Abstract

**Background:**

Perrault Syndrome (PRLTS) is a rare, autosomal recessive disorder that presents with bilateral sensorineural hearing loss in all patients and gonadal dysfunction in females. It has been linked to variants in *CLPP*, *ERAL1*, *HARS2*, *HSD17B4*, *LARS2*, and *TWNK* genes. All reported cases due to *TWNK* variants have included neurologic features, such as ataxia and axonal sensorimotor neuropathy.

**Case presentation:**

A 4.5-year-old female presented to neuromuscular clinic due to ataxia. Neurological examination revealed truncal ataxia and steppage gait, reduced deep tendon reflexes, and axonal sensorimotor polyneuropathy. Auditory brainstem response testing revealed an uncommon type of sensorineural hearing loss known as auditory neuropathy/auditory synaptopathy (AN/AS) affecting both ears. Magnetic Resonance Imaging (MRI) revealed subtle cauda equina enhancement. Nerve conduction studies led to a provisional diagnosis of chronic inflammatory demyelinating polyneuropathy (CIDP), and intravenous immune globulin (IVIG) was initiated. The patient was unresponsive to treatment, thus whole exome testing (WES) was conducted in tandem with IVIG weaning. WES revealed a compound heterozygous state with two variants in the *TWNK* gene and a diagnosis of Perrault Syndrome was made.

**Conclusions:**

Perrault Syndrome should be considered in the differential for children who present with bilateral sensorineural hearing loss, axonal polyneuropathy, and ataxia. Further examination includes testing for ovarian dysgenesis and known PRLTS genetic variants.

## Background

Perrault syndrome (PRLTS) is a rare autosomal recessive disorder, with approximately 100 affected individuals reported. These individuals present with bilateral sensorineural hearing loss (SNHL), while females (46, XX karyotype) also present with ovarian dysfunction [1, 2]. Most cases of PRLTS have unidentified genetic etiologies, though some cases are caused by biallelic pathogenic variants in one of the following genes: *CLPP*, *ERAL1*, *HARS2*, *HSD17B4*, *LARS2*, and *TWNK*, all of which are integral in mitochondrial functions [2]. The *CLPP* gene (on chromosome 19q13) encodes a subunit of the ClpP complex, which plays a role in abnormal protein breakdown in the mitochondria [3]. The *ERAL1* gene (on chromosome 17q11) encodes a GTPase protein, important for proper assembly of the mitochondrial ribosome [4]. The *HARS2* (on chromosome 5q31) and *LARS2* genes (on chromosome 3p21) are involved in translation of mitochondrial proteins [5, 6]. The *HSD17B4* gene (chromosome 5q23) is involved in beta-oxidation of fatty acids and steroid metabolism [7]. *TWNK* (on chromosome 10q24) encodes the Twinkle protein, which is a mitochondrial DNA helicase and is required for mtDNA replication [2]. It is, however, important to note that these pathogenic genes are not exclusive to PRLTS. Some of these genes have a causative relationship to other diseases, such as severe peroxisomal disorders [8]. This genetic heterogeneity makes it hard to understand a common pathophysiology. Further, while the six genes linked to PRLTS all disrupt mitochondrial processes, there are many other genes that alter mitochondrial function that are not implicated in PRLTS. This further elucidates that the pathophysiology of PRLTS is not easily understood from genetic findings alone. One must look at both the clinical and genetic picture to arrive at a PRLTS diagnosis.

Reported cases of PRLTS associated with *CLPP* and *LARS2* variants have presented with or without neurologic features, while reported cases of PRLTS due to *TWNK* variants always include neurologic features [2]. Gotta et al. recently reported the most common neurologic features in *TWNK*-related PRLTS cases include nystagmus, positive Romberg sign, ataxic gait, reduced deep tendon reflexes in lower limbs, pes cavus, and an axonal sensorimotor neuropathy [9]. Of the patients reported to have PRLTS associated with *TWNK* variants, onset of symptoms can range from < 3–48 years old [1, 9–16]. Because neuropathy is a salient feature in TWNK-associated PRLTS, this may lead to misdiagnosis of other neuropathy-associated diseases, especially in younger patients. In this study, we report a patient with PRLTS who is compound heterozygous for novel *TWNK* gene variants, review previously published TWNK-associated PRLTS cases, and highlight the clinical overlap between PRLTS and other neuropathies.

## Case presentation

A 4.5-year-old female presented to the neuromuscular clinic at Ann & Robert H. Lurie Children’s Hospital in Chicago with an abnormal gait. She was born at 37 weeks gestation in a non-consanguineous union to parents of Asian descent, without any pregnancy or delivery complications. She has a healthy 12-year-old sister. She had an age-appropriate developmental trajectory in the first year of her life. She had an upper respiratory illness at 10 months age during a visit to China after which the parents noted that she slowed down in her motor developmental trajectory. Parents’ concern was deepened when they noticed an unsteady gait after she started walking at 16 months of age. She could walk and run but would appear unsteady, leading to occasional falls. Toe walking was noted at 2 years of age, in addition to a steppage gait. Around 3 years of age she had a viral illness, after which she was noted to be more unsteady, resulting in at least 2 falls per week. Parents reported they had no concerns about speech and language development, and she was able to answer questions and follow direction in English and Mandarin until age 3.5 years. At that point she stopped responding to her name and music, following verbal instructions, and speech began to regress. Family history was non-contributory. Her weight was at the 43rd and height at 29th percentile for age. Exam was notable for a cooperative child who was inconsistent following commands, with a limited verbal output for age. There was no note of scoliosis or joint hyperextensibility and the cardiac, pulmonary, gastrointestinal exams were normal. She recognized mother and was able to point to her nose inconsistently on command. Auditory exam demonstrated inconsistencies in hearing. Extraocular movements were full and there was no nystagmus. Fundus exam did not reveal any abnormalities. The rest of the cranial nerve exam was normal. Muscle bulk was typical for age and there was absence of muscle atrophy. She had mild tightness in bilateral heel cords but otherwise tone was normal in other areas. Strength examination was limited due to cooperation, but within the limitations she was noted to have difficulty with bilateral foot eversion (4-/5) and demonstrated good power in the rest of the muscles of the face, neck, trunk, upper and lower extremities. Deep tendon reflexes were absent at the ankles and knees bilaterally and were 1 + at the other sites. She had normal coordination in her upper extremities but demonstrated a wide based, unstable and steppage quality when walking. Her Romberg’s testing was negative, suggesting that she had a form of sensory ataxia. An electromyography (EMG) and nerve conduction study demonstrated absent upper (median, ulnar and radial) and lower extremity (superficial peroneal and medial plantar) sensory responses with sparing of the sural sensory response. There was a severe decrease in lower extremity compound muscle action potential (CMAP) amplitudes in the nerves of lower extremity (Peroneal and tibial) and mild decrease in the upper extremity (median and ulnar) with normal distal latency and conduction velocities. F wave was absent in the tibial nerve. EMG demonstrated a neurogenic pattern with increased duration of voluntary motor unit potentials in the tibialis anterior and the gastrocnemius muscles. The overall picture was suggestive of a chronic axonal sensorimotor polyneuropathy. Auditory brainstem response testing was consistent with bilateral auditory neuropathy/auditory synaptopathy (also referred to as auditory dyssynchrony or auditory neuropathy spectrum disorder), hearing loss characterized by abnormal synchronization of neuronal responses to sound at the level of the auditory nerve and brainstem. Behavioral auditory evaluation of thresholds revealed profound loss. MRI of the spine (at age 4.5 years) was notable for subtle cauda equina enhancement. MRI of the brain (at age 4.5 years), internal auditory canals and cochleae was unremarkable. Creatine kinase, lactate, and cerebrospinal fluid (CSF) studies were all within normal limits. A comprehensive neuropathy panel and frataxin repeats testing was normal. On a subsequent visit she was noted to be more unsteady and, per parents, falling more frequently. IVIG was initiated, with a provisional diagnosis of CIDP based on history and cauda equina enhancement on imaging. Audiology assessments confirmed profound hearing loss and cochlear implant candidacy evaluation was initiated. She received 3 initial doses of IVIG and her parents noted slight improvement in gait. Thus, treatment was increased to every 2 weeks for 3 treatments, followed by every 3 weeks for 3 treatments. Further clinically significant improvement was not observed. Therefore, a whole exome testing (WES) was sent while IVIG weaning was initiated. WES, performed at GeneDx, Inc. using the next generation sequencing on an Ilumina platform, revealed a compound heterozygous state with two variants in the *TWNK* gene (NM_021830.4): c. 561_563dupTGA, p.Asp188dup in exon 1 and c.1909 C > T, p.Arg637Trp in exon 5. Family studies showed that the patient inherited the p.Asp188dup variant from mother and the p.Arg637Trp variant from father. Both parents were unaffected.

She underwent cochlear implantation and began a program of intensive auditory and spoken language habilitation. Sound detection thresholds improved to the normal range in both ears. However, improvement in measurable word recognition skills and spoken language were slow to develop and remained limited after one year of implant experience. Such slow progress is not typical of implanted children with a history of auditory experience and spoken language, including those with auditory neuropathy.

Since PRLTS due to *TWNK* gene variants can also cause ovarian dysfunction, further testing was done. Follicle stimulating hormone (FSH) level was 15.8 mIU/ml (reference range 1–5 mIU/ml), with normal luteinizing hormone (LH), creatine kinase (CK), and lactate levels. Ultrasound (US) of the pelvis was significant for an absence of ovarian structures. Based on the results of the clinical features of ataxia, bilateral sensorineural hearing loss, axonal sensorimotor polyneuropathy, WES results, FSH levels, and US of the pelvis, a diagnosis of PRLTS was made.

## Discussion and conclusions

We report a case of early-onset ataxia, axonal polyneuropathy, and bilateral auditory neuropathy/auditory synaptopathy, an uncommon type of sensorineural hearing loss, associated with PRLTS attributed to compound heterozygous variants in the *TWNK* gene. Our patient presented with ataxia at 16 months of age, making her onset of neurological features the youngest of all reported *TWNK*-associated PRLTS cases to date (Table 1) [1, 9–16]. This patient was initially suspected to have CIDP based on clinical presentation, MRI findings, and sensorimotor polyneuropathy on neurophysiology. IVIG treatment was initiated for a presumptive diagnosis of CIDP; however, the patient was unresponsive. After confirmation of compound heterozygosity for variants in the *TWNK* gene, IVIG was weaned off, as there is no available evidence that IVIG is effective in treating PRLTS.

About 15% of children identified with hearing loss following referral from newborn hearing screening have auditory neuropathy/auditory synaptopathy [17, 18]. Its incidence has been reported to be 24% and higher in premature infants and infants in the neonatal intensive care unit. Hearing thresholds may range from normal to profound hearing loss [19, 20]. Those affected have greater difficulty understanding spoken language, especially in the presence of noise, compared to individuals with typical sensorineural hearing loss with similar auditory thresholds. Site of lesion may be at the level of the inner hair cell, synapse of the inner hair cell and auditory nerve, and/or abnormality of the auditory nerve itself. The etiology may be acquired or genetic. Acquired forms may be related to prematurity, elevated bilirubin, hypoxia, viruses including measles, mumps and cytomegalovirus, and head trauma. Genetic forms may be nonsyndromic or syndromic. An example of a nonsyndromic and synaptic form is OTOF-Related deafness [21]. Syndromic postsynaptic forms include hereditary sensory neuropathies such as Charcot-Marie-Tooth disease and Friedrich Ataxia [22].

**Table 1:** Clinical features of TWNK-Associated PRLTS

	Sex	PRLTS Symptom Onset	Ataxia	Axonal Polyneuropathy	Nystagmus	Reduced DTR	Romberg +	Pes Cavus	SNHL	Gonad Dysfunction	FSH	Pelvic Ultrasound
**Our Patient**	F	16 months	Yes	Yes	No	Yes	No	No	Yes	Yes	Elevated	Absence of ovarian structures
**Gotta et al. 2020** [9]	F	5 YO	Yes	Yes	Yes	Yes	Yes	Yes	Yes	Yes	NR	Normal sized anteverted uterus and small ovaries
**Kume at el. 2020** [10]	F	48 YO	Yes	Yes	Yes	Yes	Yes	NR	Yes	NR	NR	NR
**Dominguez-Ruiz et al. 2019** [11]	F	5 YO	Yes	Yes	Yes	Yes	Yes	Yes	Yes	Yes	NR	NR
**Dominguez-Ruiz et al. 2019** [11]	F	3 YO	Yes	Yes	NR	Yes	NR	Yes	Yes	Yes	NR	NR
**Dominguez-Ruiz et al. 2019** [11]	M	4 YO	Yes	Yes	Yes	Yes	Yes	Yes	Yes	NR	NR	NR
**Fekete et al. 2019** [12]	F	4 YO	Yes	Yes	Yes	No (increased)	NR	Yes	Yes	Yes	Elevated	Ovarium agenesis and uterus hypoplasia
**Jamali et al. 2019** [13]	M	4 YO	Yes	Yes	Yes	NR	NR	NR	Yes	NR	NR	NR
**Demain et al. 2017** [14]	F	< 3 YO	Yes	Yes	Yes	Yes	NR	NR	Yes	Yes	NR	Small uterus and ovaries
**Oldak et al. 2017** [15]	F	3 YO	Yes	Yes	Yes	Yes	Yes	Yes	Yes	Yes	Elevated	Hypergonadotropic hypogonadism, streak gonads, rudimentary uterus
**Oldak et al. 2017** [15]	F	11 YO	Yes	Yes	Yes	Yes	Yes	Yes	Yes	Yes	Elevated	Ovarian Dysgenesis
**Lerat et al. 2016** [1]	F	< 3 YO	Yes	Yes	NR	NR	NR	NR	Yes	Yes	NR	NR
**Lerat et al. 2016** [1]	F	< 3 YO	Yes	Yes	NR	NR	NR	NR	Yes	Yes	NR	NR
**Lerat et al. 2016** [1]	M	< 3 YO	Yes	Yes	NR	NR	NR	NR	Yes	No	NR	NR
**Morino et al. 2014** [16]	F	13 YO	Yes	Yes	Yes	Yes	Yes	Yes	Yes	Yes	Elevated	NR
**Morino et al. 2014** [16]	F	8 YO	Yes	Yes	Yes	Yes	Yes	Yes	Yes	Yes	Elevated	NR
**Morino et al. 2014** [16]	F	7 YO	Yes	Yes	Yes	Yes	Yes	NR	Yes	Yes	NR	Streak ovaries
**Morino et al. 2014** [16]	F	7 YO	Yes	Yes	NR	Yes	Yes	NR	Yes	Yes	NR	Streak ovaries

*YO* Years Old, *DTR* Deep tendon reflexes, *NR* Not reported, PRLTS symptom onset is based upon age of ataxia or SNHL

Although most reported cases of *TWNK*-related PRLTS had nystagmus, pes cavus, and a positive Romberg test (Table 1), these features were not noted in our patient over the course of her evaluation [1, 9–16]. However, given our patient’s early onset of symptoms, one could postulate that they may develop these findings and, therefore, should be routinely monitored for such. This will include annual neurology follow-up visits at the neuromuscular multidisciplinary clinic, including complete neurological examination and close monitoring for any clinically significant changes. Repeat EMG and nerve conduction studies may be indicated to monitor the patient’s polyneuropathy. Additionally, the patient will continue to follow-up with Otolaryngology, continuing to work on development of measurable word recognition and spoken language skills.

Table 2 compares *TWNK-*related PRLTS to CIDP and Friedreich’s Ataxia, all of which can present with sensorimotor neuropathy, ataxia, and reduced to absent deep tendon reflexes. PRLTS due to *TWNK* variants and Friedreich’s Ataxia can additionally present with nystagmus and sensorineural hearing loss, which is rare in CIDP. However, only *TWNK-*related PRLTS commonly presents with gonadal dysfunction. Thus, we suggest that children who present with the clinical signs of ataxia and polyneuropathy should not only be evaluated for CIDP and Friedreich’s Ataxia, but also for PRLTS, specifically evaluating gonadal function in females and genetic testing for known variants. Additionally, when considering these differential diagnoses, careful attention should be paid to the presence of sensorineural hearing loss and CSF findings. As previously discussed, CIDP rarely presents with sensorineural hearing loss. CIDP additionally presents with elevated CSF protein in most cases, whereas our patient’s CSF findings were all within normal limits. Thus, the presence of sensorineural hearing loss and normal CSF protein supports the diagnosis of PRLTS versus an acquired neuropathy, such as CIDP.

**Table 2:** Differential Diagnosis for Childhood Ataxia with Sensorimotor Polyneuropathy

	*TWNK*-related PRLTS	CIDP	Friedreich’s Ataxia
**Sensorineural HL**	+	Rare	+
**Nystagmus**	+	Rare	+
**Pes Cavus**	+	-	+
**Reduced DTR’s**	+	+	+
**Positive Romberg sign**	+	+	+
**Ataxia**	+	+	+
**Axonal Sensorimotor polyneuropathy**	+	+	+
**Gonadal Dysfunction**	+	-	-
**Improvement with IVIG**	-	+	-

Genetic testing for our patient demonstrated two variants (Table 3). The p.Asp188dup leads to an in-frame insertion of one amino acid and TGA duplication. This change has not been observed in population cohorts nor in affected individuals to our knowledge. The p.Arg637Trp missense variant is predicated to have a deleterious effect on the protein function via in silico analysis. To our knowledge, the variant has not been reported previously in affected individuals. The p.Arg637Trp variant has been observed in 0.0007% in large population cohorts [23]. Both variants were interpreted and classified as uncertain clinical significance at this time.

**Table 3:** Reported TWNK Variants in PRLTS Cases

	Sex	Ancestry	Genetic variants TWNK (NM_021830)
**Our Patient**	Female	Asian	c.561_563dupTGA (p.Asp188dup) + c.1909 C > T (p.Arg637Trp)
**Gotta et al. 2020** [9]	Female	Italian	c.743T > C (p.Phe248Ser) + c.1519G > A (p.Val507Ile)
**Kume at el. 2020** [10]	Female	Japanese	homozygous c.1358G > A (p. Arg453Gln)
**Dominguez-Ruiz et al. 2019** [11]	Female	Spanish	c.85 C > T (p.Arg29*) + c.1886 C > T (p.Ser629Phe)
**Dominguez-Ruiz et al. 2019** [11]	Female	Spanish	c.85 C > T (p.Arg29*) + c.1886 C > T (p.Ser629Phe)
**Dominguez-Ruiz et al. 2019** [11]	Male	Spanish	c.85 C > T (p.Arg29*) + c.1886 C > T (p.Ser629Phe)
**Fekete et al. 2019** [12]	Female	Hungarian	c.1196 A > G, (p.Asn399Ser) + c.1358G > A (p. Arg453Gln)
**Jamali et al. 2019** [13]	Male	Iranian	homozygous c.874 C > A (p.Pro292Thr)
**Demain et al. 2017** [14]	Female	Norwegian	c.968G < A (p.Arg323Gln) + c.1196 A > G (p.Asn399Ser)
**Oldak et al. 2017** [15]	Female	Polish	c.1196 A > G (p.Asn399Ser) + c.1802G > A (p.Arg601Gln)
**Oldak et al. 2017** [15]	Female	Polish	c.1196 A > G (p.Asn399Ser) + c.1802G > A (p.Arg601Gln)
**Lerat et al. 2016** [1]	Female	Moroccan	homozygous c.793 C > T (p.Arg265Cys)
**Lerat et al. 2016** [1]	Female	Moroccan	homozygous c.793 C > T (p.Arg265Cys)
**Lerat et al. 2016** [1]	Male	Moroccan	homozygous c.793 C > T (p.Arg265Cys)
**Morino et al. 2014** [16]	Female	Japanese	c.1172G > A (p.Arg391His) + c.1754 A > G (p.Asn585Ser)
**Morino et al. 2014** [16]	Female	Japanese	c.1172G > A (p.Arg391His) + c.1754 A > G (p.Asn585Ser)
**Morino et al. 2014** [16]	Female	Greek	c.1321T > G (p.Trp441Gly) + c.1519G > A (p.Val507Ile)
**Morino et al. 2014** [16]	Female	Greek	c.1321T > G (p.Trp441Gly) + c.1519G > A (p.Val507Ile)

Previously reported literature and our case shows a predominance of females, although the inheritance is autosomal recessive. We postulate that this could be due to an increased recognition of PRLTS in females with ovarian dysfunction, adding to the phenotype of sensorineural hearing loss and ataxia. It has been claimed that PRLTS is not as rare as would be expected from the paucity of reported cases [9, 11]. This may be true and we hypothesize that there may be a cohort of children who have been diagnosed as having either sensorineural hearing loss or ovarian dysfunction, who have not been evaluated for sensorimotor polyneuropathy or found to have a combination of the above, who could have PRLTS. We, therefore, recommend that audiology, ENT, OBGYN, endocrinology, and adolescent medicine providers evaluating children with either hearing loss or ovarian dysfunction actively inquire regarding symptoms of neuropathy, such as tingling, numbness, gait instability, etc. To extend the recommendations to pediatric neurology providers, Gotta et al. reported PRLTS patients with the *TWNK* variants demonstrate significant neurological deficits, yet not all previous case reports have included comprehensive neurological examinations in their findings [9]. We suggest that neurologists who encounter nystagmus, ataxia, reduced DTR’s, Romberg’s sign, and pes cavus as evidence of potential peripheral neuropathy should consider PRLTS. For the neuromuscular providers, PRLTS could be considered in the differential diagnosis for a child who presents with sensorimotor polyneuropathy and sensorineural hearing loss, who either does not meet criteria for CIDP or has symptoms refractory to IVIG [24, 25].

Finally, based on the natural history of our patient, we recommend that female children presenting with axonal polyneuropathy and ataxia should be evaluated for ovarian dysgenesis and genetic testing for PRLTS should be considered.

## Data Availability

The datasets used and/or analyzed during the current study are available from the corresponding author on reasonable request.

## Abbreviations

CIDP: Chronic inflammatory demyelinating polyneuropathy
CK: Creatine Kinase
CMAP: Compound muscle action potential
CSF: Cerebrospinal fluid
EMG: Electromyography
FSH: Follicle Stimulating Hormone
IVIG: Intravenous immune globulin
LH: Luteinizing Hormone
MRI: Magnetic Resonance Imaging
PRLTS: Perrault Syndrome
SNHL: Sensorineural hearing loss
US: Ultrasound
WES: Whole exome testing
